# Treatment Interruptions During Stereotactic Body Radiotherapy for Prostate Cancer

**DOI:** 10.3389/fonc.2021.796496

**Published:** 2022-01-19

**Authors:** Abigail N. Pepin, Alan Zwart, Malika Danner, Marylin Ayoob, Thomas Yung, Brian T. Collins, Deepak Kumar, Simeng Suy, Nima Aghdam, Sean P. Collins

**Affiliations:** ^1^ Department of Radiation Oncology, Hospital of the University of Pennsylvania, Philadelphia, PA, United States; ^2^ Department of Radiation Medicine, School of Medicine, Georgetown University, Washington, DC, United States; ^3^ Department of Radiation Medicine, MedStar Georgetown University Hospital, Washington, DC, United States; ^4^ The Julius L. Chambers Biomedical Biotechnology Research Institute, North Carolina Central University, Durham, NC, United States; ^5^ Department of Radiation Oncology, Beth Israel Deaconess Medical Center, Harvard Medical School, Boston, MA, United States

**Keywords:** SBRT (stereotactic body radiation therapy), prostate cancer, treatment interruption, Treatment delay, treatment noncompletion

## Abstract

**Background:**

During the course of radiation treatment for prostate cancer, patients may have unintentional interruptions in their treatment course due to a wide variety of factors. Stereotactic body radiation therapy (SBRT) decreases the number of treatments compared to conventionally fractionated radiation; hence, it has the potential to decrease treatment delays and non-completion. This study sought to determine the incidence of treatment delay and characterize the etiology and length in a large cohort of men treated with SBRT for their prostate cancer.

**Methods:**

One thousand three hundred and thirty-six patients treated with SBRT from 2008 to 2021 at the Georgetown University Hospital for prostate cancer were included in this retrospective study. A treatment delay was defined as a patient requiring longer than 14 days to complete 5 fractions of SBRT. Non-completion was defined as patients treated with less than 5 fractions. In the patients who experienced delays, chart review was performed to characterize the length and etiology of each delay. Multivariate analysis was performed *via* binary logistic regression modeling on PSPP.

**Results:**

All individuals in the cohort eventually completed the planned 5-fraction regimen. Thirty-three patients experienced a treatment delay. Median length of time to complete treatment was 11 days (range 5–155 days). In patients who experienced a delay, nearly half (45.5%) experienced only a one-day delay. The most common reason for a delay was a technical issue (48.5%), including the machine maintenance, fiducial misalignment, or inadequate pretreatment bowel preparation. Other reasons included unplanned breaks due to acute side effects (21.2%), logistical issues (18.2%), non-treatment related health issues (9.1%), and inclement weather (3.0%). There were no significant sociodemographic, oncologic, or treatment variables that predicted treatment interruption on multivariate analysis.

**Conclusions:**

The incidence of treatment interruptions in patients undergoing SBRT for their prostate cancer was low. Most treatment delays were short.

## Introduction

Prolongation of radiation treatment has the potential to increase tumor repopulation and affect tumor control rates (1–3). This is particularly true in patients with anal, cervical, lung, and head and neck cancers (3). In prostate cancer, which has a more indolent disease course, the results of treatment prolongation on outcomes have been mixed (1, 2).

Several retrospective studies have looked at treatment interruptions in prostate cancer patients treated with definitive external beam radiation therapy (EBRT). The University of Florida reported decreased rates of five-year local control in patients who had >8 weeks of treatment (4). However, their study was conducted in an era before PSA surveillance. A more contemporary study by D’Ambrosio et al., which examined patients treated between 1989 and 2004, demonstrated longer treatment durations as a risk factor for 10-year freedom from biochemical failure in low-risk patients (2). Dong et al. investigated the role of treatment interruptions in patients undergoing dose escalation to ≥74 Gy using IMRT or 3D-CRT and found no significant difference in outcomes with median follow up of 54 months (5). Although all these studies were performed using conventional radiation therapy, the results of treatment interruption are mixed perhaps due to the impact of total dose delivered and fractionation impacting oncologic outcomes.

The adoption of ultra-hypofractionated treatment regimens allows for decreased total treatment duration to one to two weeks. Recent studies comparing conventionally fractionated and ultra-fractionated radiation therapy have demonstrated the safety and efficacy of ultra-fractionation (6–8). As such, stereotactic body radiation therapy (SBRT) has been increasingly adopted in centers across the world. Despite this, many patients may still have unintentional treatment interruptions, which cause delays. The purpose of the current study is to evaluate rates of treatment delays and characterize the delays in a large institutional cohort of prostate cancer patients who underwent SBRT.

## Methods

### Patient Selection

The Georgetown University Institutional Review Board approved this single institutional review (IRB# 2009-510). All individuals diagnosed with prostate cancer who received SBRT at the Medstar Georgetown University Hospital from 2008 to 2021 were eligible for inclusion. Patients treated with SBRT to prostatic fossa or distant sites were excluded.

### SBRT Treatment Planning and Delivery

All men were treated with SBRT using an institutional protocol for simulation, contouring, and treatment planning (9). Patients underwent a treatment planning CT and pelvic MRI at least 1 week after placement of gold fiducial markers with or without hydrogel rectal spacers. The prescription dose was 30–37.5 Gy delivered over five fractions. The clinical target volume (CTV) included the prostate and proximal seminal vesicles. The PTV equaled the CTV expended 3 mm posteriorly and 5 mm in all other directions.

### Definitions and Statistical Analysis

In eligible patients, chart review was performed to determine date from start of treatment to end of treatment. Treatment interruptions were defined as patients requiring longer than 14 days to complete 5 fractions. In the patients who experienced delays, chart review was performed to characterize the reason for treatment delay. Causes of delay were classified as technical (i.e., mechanical failure, fiducial migration), logistical (i.e., patient caring for family member, insufficient transport), acute side effects, health issues not related to radiation treatment, and inclement weather. Patients who completed under their prescribed fraction were characterized as treatment noncompletion.

Multivariate analysis was performed using binary logistic regression on PSPP. The primary dependent variable was treatment delay. Covariables were selected based on previous investigations identifying independent determinants of SBRT use (10, 11). These included sociodemographic factors, such as race and age, oncologic factors such as Gleason scoring and stage), and also treatment variables (namely, risk grouping, SBRT dose, and ADT).

## Results

Between 2007 and May 2021, one-thousand three hundred and thirty six patients with prostate cancer were eligible for inclusion in this study. Patient characteristics are listed in **Table 1**. The patient cohort was diverse. The median age was 70 years old (range 44–100). Approximately 59% of the cohort was white and 34.4% was African American. The cohort consisted of 20% low-risk, 68.3% intermediate-risk, 11.6% high risk, and 0.07% very high-risk individuals. Seventy-six percent of patients did not receive neoadjuvant androgen deprivation therapy. Seventy-nine percent were treated with 36.25 Gy in 5 fractions, while 20.6% underwent 35 Gy in 5 fractions.

**Table 1:** Patient characteristics.

	Percent of patients (n = 1,336)
**Age (Range 44–100)**	
40–49	0.7% (10)
50–59	8.7% (116)
60–69	39.0% (521)
70–79	42.3% (565)
80–90	8.6% (115)
90+	0.7% (9)
**Race**	
African American	34.4% (459)
Caucasian	58.5% (782)
Hispanic	2.2% (29)
Other	4.9% (66)
**Gleason**	
4–5	0.45% (6)
6	29.6% (395)
7	62.0% (828)
8	5.8% (77)
9–10	2.2% (30)
**T Stage**	
T1a–T1c	68.3% (913)
T2a–T2c	30.8% (412)
T3	0.67% (9)
Tx	0.15% (2)
**D’Amico Risk Group**	
Low	20.0% (267)
Intermediate	68.3% (913)
High	11.6% (155)
Very High	0.07% (1)
**ADT**	
Yes	23.8% (318)
No	76.2% (1018)
**SBRT Dose**	
35	20.6% (275)
36.25	78.7% (1052)
Other	0.7% (9)

**Figure 1:**
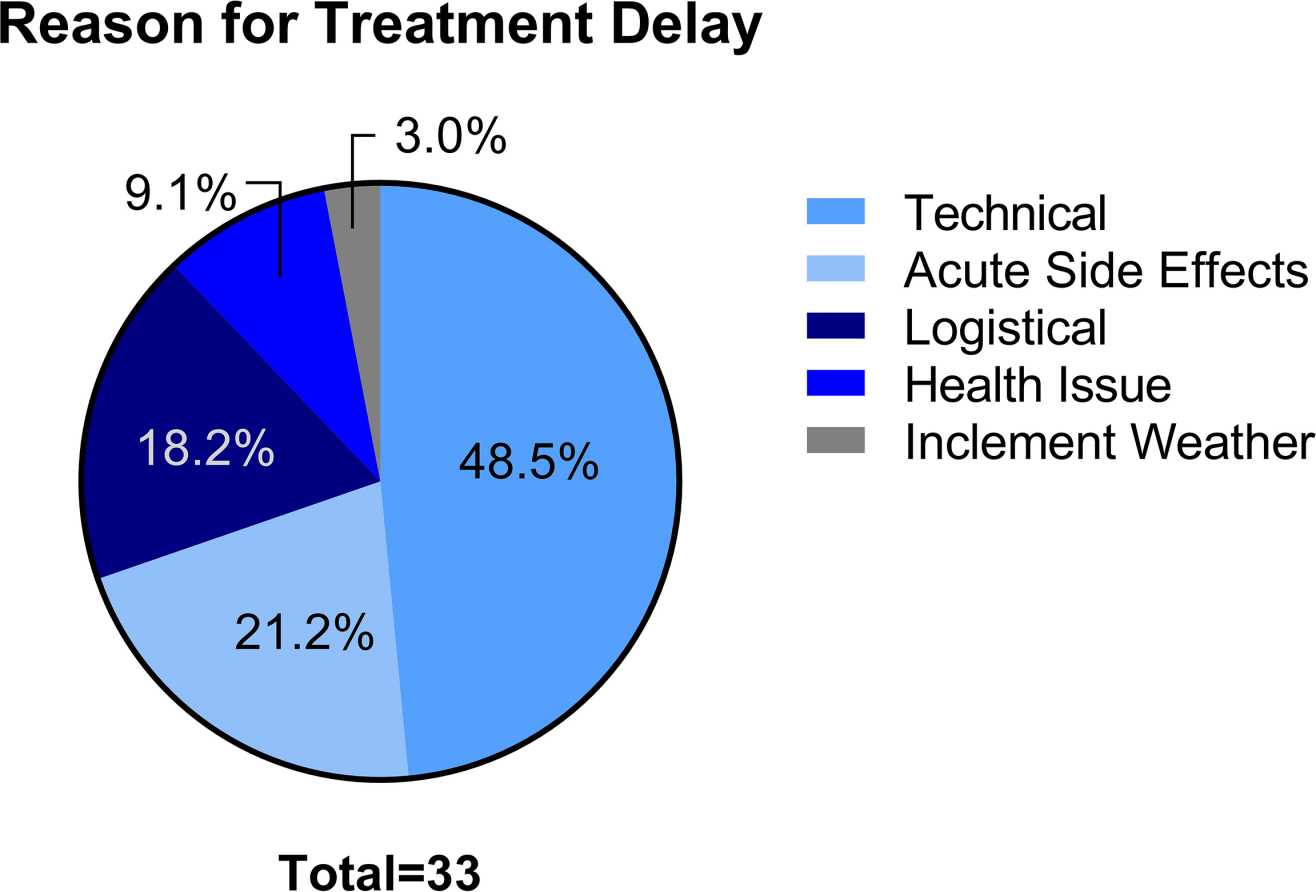
Reason for treatment delay.

**Figure 2:**
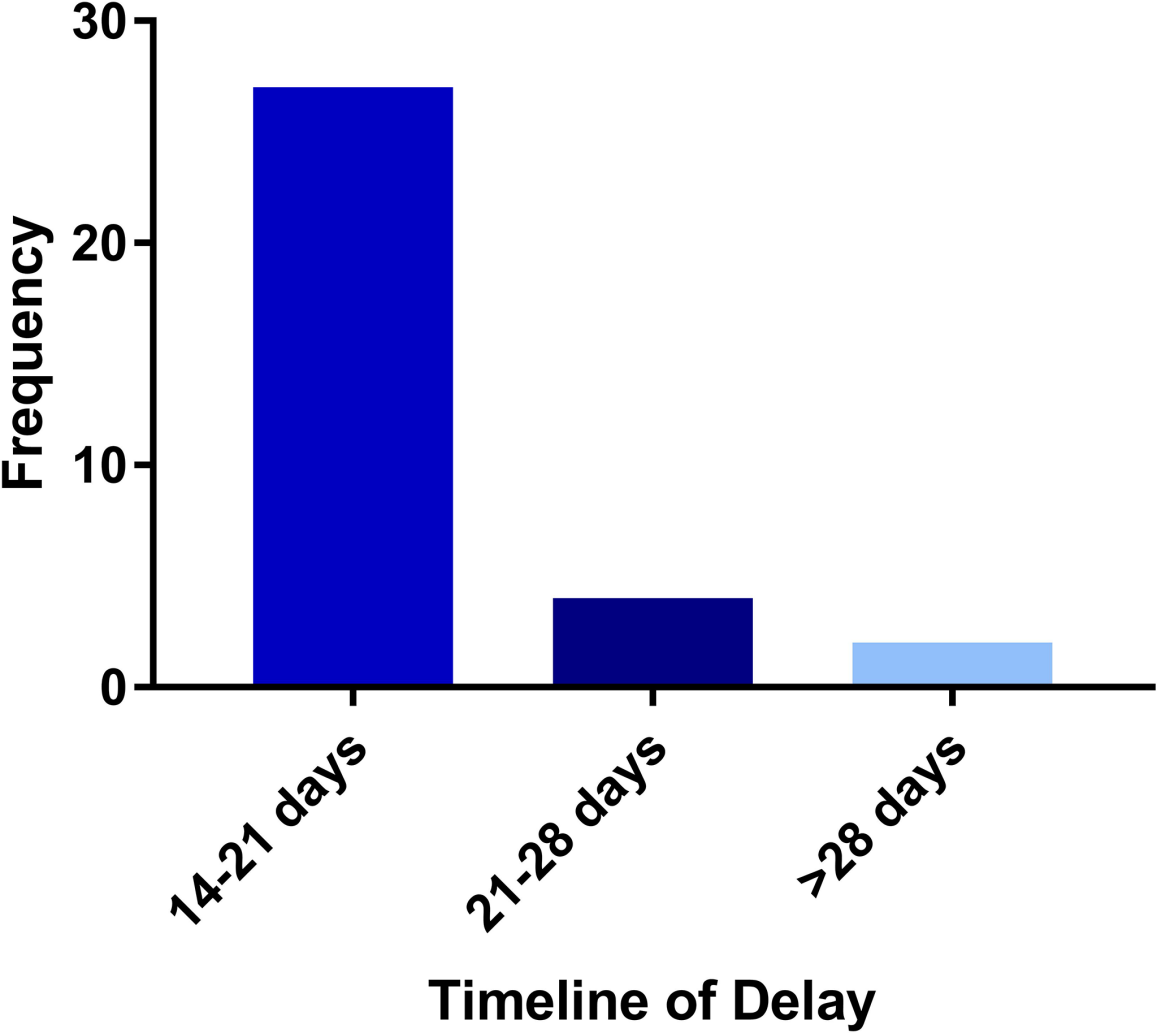
Timeline of treatment delay.

The median time for treatment completion was 11 days. Thirty-three patients (2.5%) experienced treatment delay (**Table 2**). There were no patients who experienced incompletion of treatment. The most common reason for treatment interruption was technical issues (48.5%), namely, machine downtime and fiducial migration (**Figure 1**) . Acute side effects (21.2%), logistical (18.2%), health issues not related to radiation treatment (9.1%), and inclement weather (3.0%) represented other reasons for treatment interruption. In those who experienced treatment interruption, 81.8% experienced a treatment interruption of less than or equal to one week (**Figure 2**).

**Table 2:** Length of Delay and Cause for Individual Patients.

Patient	Length of Delay (Days from Start of Treatment)	Cause
**1**	15	Technical (Machine Down)
**2**	15	Logistical Issue (No Ride)
**3**	154	Acute Side Effects (Requiring TURP)
**4**	16	Logistical Issue (Family Emergency)
**5**	14	Acute Side Effects
**6**	15	Technical (Poor Bowel Prep)
**7**	14	Acute Side Effects
**8**	15	Technical (Machine Down)
**9**	14	Technical (Machine Down)
**10**	14	Technical (Machine Down)
**11**	14	Technical (Machine Down)
**12**	15	Technical (Machine Down)
**13**	14	Technical (Machine Down)
**14**	23	Technical (Machine Down)
**15**	15	Technical (Machine Down)
**16**	14	Acute Side Effects
**17**	14	Health Issue (Arrhythmia)
**18**	43	Health Issue (Pyelonephritis)
**19**	14	Technical (Machine Down)
**20**	17	Technical (Machine Down)
**21**	15	Technical (Machine Down)
**22**	14	Logistical Issue (Dialysis)
**23**	16	Technical (Poor Bowel Prep)
**24**	23	Health Issue (COVID-19)
**25**	14	Inclement Weather
**26**	15	Acute Side effects
**27**	14	Acute Side Effects
**28**	14	Technical (Machine Down)
**29**	14	Logistical Issue (Caring for Ill Family Member)
**30**	28	Logistical Issue (No ride)
**31**	14	Acute Side Effects
**32**	18	Technical (Fiducial Migration)
**33**	24	Logistical Issue (Work Conflict)

Race, stage, ADT status, Gleason score, D’Amico risk grouping, and SBRT dose were not associated with significantly different odds for treatment interruption (**Supplementary Table 1**).

## Discussion

Our findings suggest radiation treatment interruptions and noncompletion in patients undergoing SBRT for their prostate cancer are uncommon. Our results are consistent with previously reported results which showed lower odds of treatment noncompletion in patients undergoing SBRT compared to conventionally fractionated regimens (OR 0.21) (10). The rationale for these findings are likely related to the convenience of having a smaller fraction of treatments. This is in line with the study of Han et al., which demonstrated that total treatment duration in patients undergoing proton therapy increased likelihood of treatment interruption on multivariate analysis (OR 1.05) (1).

In patients who experienced delays, many delays were unavoidable due to health concerns, patient logistical issues, or machine maintenance. This seems consistent with barriers identified in previous investigations (1). The most common cause of delay was due to technical issue. In a study investigating proton beam availability on patient treatment scheduling, it was found that machine downtime greater than 1 h may result in missed treatments (12). Because many centers have more than one linear accelerator, it is possible that treatment delays due to machine downtime may be less in patients undergoing photon-based therapy as patients can be switched between machines. In an international trial looking at linear accelerator downtime in UK, Botswana, and Nigeria facilities, downtime lasting more than 1 h was rare and occurred only 3.4% of the total faults (13). Technical delays were minimized at our center by having two robotic linear accelerators and the availability of spare parts near our center.

The overwhelming majority of the delays were ≤7 days in our study. In patients undergoing EBRT with RT doses of ≥74 Gy, slightly prolongation of treatment time (e.g., ≤7 days) was not associated with inferior freedom from biochemical failure (14). Extreme hypofractionated regimens may have mechanisms of cellular death more similar to brachytherapy than conventionally or moderately hypofractionated external beam radiation therapy; hence, it remains unclear if treatment delays would have significant impact on patient outcomes. Tamponi et al. demonstrated fraction size sensitivity was lower for prostate cancer compared to normal tissue late side-effects favoring the role of hypofractionated radiation in prostate cancer (15). A limited dependence on repopulation was observed in that study (15). Further investigations as to the impact of treatment delays in prostate cancer patients undergoing SBRT on freedom from biochemical failures are warranted.

Investigations as to optimal treatment time for SBRT are ongoing in the Patriot study with regard to biochemical failure. However, published results suggest it is safe to treat once weekly with improved quality of life scores in acute bowel and urinary scores in patients undergoing every week treatment as opposed to every other day (16).

Previous investigations have also demonstrated sociodemographic variables associated with increased rates of noncompletion and receipt of radiation therapy including younger age, black race, lower socioeconomic status, and higher risk group (10, 11). The results of our multivariate analysis failed to demonstrate age, black race, and higher risk groups as being risk factors for treatment interruption in patients undergoing SBRT for their prostate cancer.

Due to the retrospective nature of our study design, it is inherently limited. In our analysis, we have selected a number of covariates based on studies conducted from the National Cancer Database (NCDB) (10, 11). There are a number of studies documenting limitations of using the NCDB, namely, selection bias, lack of clinically relevant endpoints, and prevalence of missing data among hospital-based cancer patients (17, 18). Despite this, we believe that the results of our analysis may have been negative as nearly half of our patients who experienced delays were due to technical issues, which would be independent of sociodemographic factors.

## Conclusion

The incidence of treatment interruptions in patients undergoing SBRT for their prostate cancer was low. Most treatment delays were short.

## Data Availability Statement

The datasets will not be made available due to patient privacy concerns. Requests to access the datasets should be directed to SPC9@gunet.georgetown.edu.

## Author Contributions

AP was the lead author, who participated in data collection, data analysis, manuscript drafting, table/figure creation, and manuscript revision. AZ and MD participated in data collection and data analysis while also aiding in study design. TY and MA aided in clinical data collection. DK participated in data analysis and manuscript review. BC aided in manuscript review. SS is a senior author who organized the data and participated in its analysis. NA is a senior author who aided in data analysis and manuscript review and revision. SC was the principal investigator who initially developed the concept of the study and the design, aided in data collection, and drafted and revised the manuscript. All authors contributed to the article and approved the submitted version.

## Funding

The Department of Radiation Medicine at Georgetown University Hospital receives a grant from Accuray to support a research coordinator. This work was supported by The James and Theodore Pedas Family Foundation. SC and DK acknowledge the grant R01MD012767 from the National Institute on Minority Health and Health Disparities.

## Conflict of Interest

The Department of Radiation Medicine at Georgetown University Hospital receives a grant from Accuray to support a research coordinator. BC and SC serve as clinical consultants to Accuray.

The remaining authors declare that the research was conducted in the absence of any commercial or financial relationships that could be construed as a potential conflict of interest.

## Publisher’s Note

All claims expressed in this article are solely those of the authors and do not necessarily represent those of their affiliated organizations, or those of the publisher, the editors and the reviewers. Any product that may be evaluated in this article, or claim that may be made by its manufacturer, is not guaranteed or endorsed by the publisher.

## Supplementary Material

Supplementary Table 1 Rates of treatment interruption and odds ratios stratified by sociodemographic and oncologic variables.
