# Supplementary material for: Treatment Interruptions During Stereotactic Body Radiotherapy for Prostate Cancer
Source: Front Oncol. 2022 Jan 19;11:796496. doi: 10.3389/fonc.2021.796496 (PMC8807506; doi:10.3389/fonc.2021.796496)
Supplement: Supplementary Table 1 — Rates of treatment interruption and odds ratios stratified by sociodemographic and oncologic variables. [file DataSheet_1.docx]

**Supplemental Table 1. Rates of treatment interruption and odds ratios stratified by sociodemographic and oncologic variables**

|  | **% (n) who experienced delays** | **OR for treatment interruption** | **95% CI** | ***P-value*** |
| --- | --- | --- | --- | --- |
| **Age** | NA | 0.98 | 0.93-1.03 | 0.25 |
| **Race**  White  Black  Other | 2.43% (19)  2.18% (10)  4.21% (4) | Ref  0.81  1.62 | -  0.36-1.82  0.53-4.96 | -  0.60  0.40 |
| **Stage**  T1  T2  T3  Tx | 2.52% (23)  2.43% (10)  0% (0)  0% (0) | Ref  0.80  0.00  0.00 | -  0.36-1.77  0.00- ∞  0.00- ∞ | -  0.58  0.99  1.00 |
| **Gleason**  4  5  6  7  8  9  10 | 0% (0)  0% (0)  2.28% (9)  2.22% (18)  5.19% (4)  7.69% (2)  0% (0) | 0.00  0.00  1.06  Ref  2.01  3.52  0.00 | 0.00-∞  0.00-∞  0.31-3.62  -  0.24-16.97  0.32-39.2  0.00-∞ | 1.00  1.00  0.93  -  0.51  0.30  1.00 |
| **Risk**  Low  Intermediate  High  Very High | 2.22% (6)  2.19% (20)  4.52% (7)  0% (0) | Ref  1.02  0.95  0.00 | -  0.25-4.21  0.09-9.79  0.00-∞ | -  0.98  0.97  1.00 |
| **ADT**  Yes  No | 4.09% (13)  1.96% (20) | Ref  0.53 | -  0.23-1.20 | -  0.13 |
| **SBRT dose** | NA | 0.96 | 0.50-1.84 | 0.90 |
